# Translational Development of Physiologic IGF-1 Replacement for the Prevention of Bronchopulmonary Dysplasia

**DOI:** 10.3390/biomedicines14081871

**Published:** 2026-08-21

**Authors:** Victoria Niklas, Norman Barton

**Affiliations:** Oak Hill Bio Holdings Ltd., Altrincham WA14 2DT, UK; norman.barton@sirogenesis.com

**Keywords:** bronchopulmonary dysplasia, extreme prematurity, insulin-like growth factor 1, IGF binding protein 3, developmental therapeutics, developmental biology, neonatal drug development, translational medicine

## Abstract

Bronchopulmonary dysplasia (BPD) remains the most common chronic respiratory complication of extremely preterm infants despite major advances in neonatal intensive care. Although contemporary therapies have improved survival by reducing secondary lung injury, they have had limited impact on the disrupted lung development that characterizes modern BPD. This has renewed interest in developmental therapeutics that seek to restore physiologic signaling pathways interrupted by premature birth rather than treat established disease. Among the developmental pathways disrupted by premature birth, observational studies consistently demonstrate that low postnatal IGF-1 concentrations in extremely preterm infants are associated with an increased risk of BPD and other complications of prematurity. Although these associations do not establish causality, they provide a biologically plausible basis for investigating physiologic IGF-1 replacement. This review summarizes the developmental biology of the IGF axis and the translational pathway supporting the clinical development of OHB-607, a recombinant human IGF-1/IGF-binding protein-3 complex intended to restore physiologic IGF-1 concentrations in extremely preterm infants at high risk for BPD. The program provides one example of how developmental biology, translational animal models, developmental pharmacology, and clinical implementation can be integrated into a framework for advancing mechanism-based therapies aimed at preserving normal lung development after extremely premature birth. Whether this approach improves clinically meaningful outcomes remains to be determined in adequately powered randomized clinical trials.

## 1. Introduction

Bronchopulmonary dysplasia (BPD) remains the most common pulmonary complication after extremely preterm birth (<28 weeks’ gestation) and the leading cause of chronic lung disease in these infants [1]. Despite major advances in neonatal intensive care (NICU), including surfactant replacement, non-invasive respiratory support, and optimized nutrition, the incidence of severe BPD has changed little over the past decades [2,3]. Because extremely preterm infants are born during the late canalicular and saccular stages of lung development, alveolar and pulmonary vascular maturation remain incomplete. Consequently, contemporary or “new” BPD is now recognized as a disorder of disrupted lung development rather than simply a consequence of ventilator-induced injury [1,4,5].

This developmental phenotype differs fundamentally from the “old” BPD originally described in relatively more mature preterm infants who survived prolonged exposure to invasive ventilation and hyperoxia [6,7,8]. The clinical consequences of “new” BPD extend well beyond the neonatal period, with affected infants experiencing prolonged respiratory support, recurrent respiratory morbidity, adverse neurodevelopment, and higher mortality throughout childhood and beyond [9,10,11,12]. Although improvements in neonatal care have substantially increased survival, they have had only a modest impact on the incidence and severity of BPD and its impact on healthcare utilization and the infant and family [13,14,15].

Current preventive and therapeutic strategies, including optimized respiratory support, corticosteroids, caffeine, vitamin A, and nutritional interventions, primarily reduce secondary injury rather than directly address the disrupted developmental biology underlying BPD [5,16,17,18]. Systemic corticosteroids facilitate extubation and reduce pulmonary inflammation in infants at highest risk but are used selectively because of concerns regarding adverse neurodevelopmental outcomes [18,19,20]. Likewise, caffeine improves respiratory outcomes and reduces the incidence of BPD, although its benefit varies across gestational age and clinical risk groups [21,22]. Collectively, these therapies improve neonatal outcomes but do not restore the developmental signaling pathways interrupted by premature birth.

This evolving understanding has shifted attention toward the developmental origins of BPD. Premature birth not only exposes the immature lung to postnatal injury but also abruptly interrupts the growth and other signaling pathways that normally regulate pulmonary development throughout the third trimester [5,16,23,24,25]. Developmental therapeutics has emerged as one complementary approach that seeks to restore physiologic signaling pathways disrupted by premature birth rather than treating the downstream consequences of established injury. As emphasized in the Lancet Child & Adolescent Health Commission on the Future of Neonatology review, this approach may complement conventional supportive care through mechanism-based interventions targeting the developmental origins of disease [16,26].

This review examines the developmental biology of the IGF axis, the translational evidence supporting physiologic IGF-1 replacement, and the scientific and practical considerations underlying mechanism-based therapies for BPD [23,24,27,28,29]. OHB-607, a recombinant human IGF-1/recombinant human IGF binding protein-3 (rhIGF-1/rhIGFBP-3) complex, is discussed as one example of a developmental therapeutic strategy intended to restore circulating IGF-1 concentrations toward the physiologic fetal range after extremely preterm birth. Throughout this review, OHB-607 is presented as an illustrative example of developmental therapeutics rather than as evidence of definitive clinical efficacy. The available preclinical, translational, and early clinical data provide a biologically plausible basis for continued investigation, while confirmation of clinical benefit awaits adequately powered randomized trials [30,31,32,33,34,35,36,37,38].

## 2. Developmental Therapeutics: Biological Rationale for Physiologic IGF-1 Replacement

IGF-1 is a key fetal growth factor that supports organ growth and maturation during the third trimester [39]. In the developing lung, IGF-1 promotes pulmonary angiogenesis, alveolarization, and coordinated vascular development through interactions with vascular endothelial growth factor (VEGF) and other developmental signaling pathways [27,36,40,41].

During fetal life, circulating IGF-1 is supplied predominantly through the maternal–placental unit until approximately 30 weeks’ gestation, when endogenous hepatic production becomes increasingly growth hormone dependent [39,42]. Consequently, birth before 28 weeks’ gestation results in an abrupt decline in circulating IGF-1 concentrations to levels well below those of gestational age-matched fetuses in utero, creating a prolonged period of physiologic IGF-1 deficiency that often persists until approximately 30 weeks’ postmenstrual age [28,36,38,39,43].

Persistent postnatal IGF-1 deficiency has been associated with impaired pulmonary microvascular development, arrested alveolarization, alveolar simplification, and reduced capillary growth, hallmarks of “new” BPD [36,44]. Beyond the lung, low circulating IGF-1 has also been associated with other complications of extreme prematurity, including retinopathy of prematurity (ROP), intraventricular hemorrhage (IVH), necrotizing enterocolitis (NEC), and adverse neurodevelopmental outcomes [7,27,29,36,42].

Collectively, these observations provide a biologically plausible basis for investigating physiologic IGF-1 as a developmental therapeutic strategy. However, these associations do not establish causality, and whether restoration of physiologic IGF-1 concentrations improves pulmonary or multisystem outcomes remains to be determined in adequately powered clinical trials.

## 3. Preclinical Development: Translational Evidence Supporting Physiologic IGF-1 Replacement

The preclinical development of OHB-607 was designed to translate developmental biology into a clinically feasible therapeutic strategy. Rather than following a conventional dose-escalation paradigm, the program sought to determine whether restoring circulating IGF-1 concentrations toward the physiologic fetal range could support normal lung development after extremely preterm birth. Because no single experimental model reproduces the complexity of extreme prematurity or neonatal intensive care, complementary mechanistic, translational, and pharmacologic studies were undertaken to establish biologic plausibility, optimize developmental exposure, and support clinical investigation.

Initial studies defined the developmental role of IGF-1 during lung morphogenesis. Rodent models demonstrated that impaired IGF signaling disrupts alveolar septation, pulmonary angiogenesis, elastin organization, and distal airspace formation, whereas restoration of IGF activity has been associated with preservation of alveolar complexity and pulmonary vascular development in experimental models [27,32]. These findings established IGF-1 as a critical regulator of coordinated vasculo-alveolar development and provided the mechanistic rationale for subsequent translational studies.

To evaluate these observations under conditions more representative of neonatal intensive care, investigation progressed to preterm lamb models (Table 1). Because respiratory support, surfactant replacement, vascular access, nutritional management, and pulmonary physiology closely resemble those of extremely preterm infants, this model provided an important bridge between experimental biology and clinical translation. Continuous infusion of the rhIGF-1/rhIGFBP-3 complex in the preterm lamb model was associated with restoration of circulating IGF-1 concentrations toward the fetal physiologic range and with improvements in lung compliance, oxygenation, pulmonary vascular development, capillary density, and alveolarization without evidence of abnormal tissue overgrowth [34,35]. These data provided essential proof of concept support during review by regulatory authorities.

The translational findings from these studies also informed developmental pharmacology. Rather than maximizing systemic exposure, the objective was to restore physiologic IGF-1 concentrations during a critical developmental window [34,35]. Pharmacokinetic studies, performed in the preterm lamb model of BPD and modeled after studies in humans, demonstrated that the short circulating half-life of free IGF-1 required continuous intravenous infusion to maintain stable physiologic exposure. Population pharmacokinetic modeling integrated preclinical and early clinical data to define exposure targets that informed clinical dose selection [31,38]. Although preterm lambs provide one of the closest available models of extremely preterm human lung development, they cannot fully reproduce the complexity of human neonatal intensive care or the biological heterogeneity of extremely premature infants.

Taken together, mechanistic studies, translational large-animal investigations, and developmental pharmacology support progression to clinical investigation, providing complementary evidence for a translational framework for biological plausibility and feasibility of physiologic IGF-1 replacement (Table 1). Nevertheless, these experimental models cannot predict clinical efficacy, and confirmation of therapeutic clinical benefit requires adequately powered clinical trials.

## 4. Clinical Translation: Developmental Pharmacology and Clinical Implementation

Translating physiologic IGF-1 replacement into clinical investigation involved a developmental pharmacology strategy that differs from conventional drug development. Pharmacokinetics in infants born before 28 weeks’ gestation are influenced by rapid physiologic maturation, including gestational and postmenstrual age, organ development, birth weight, nutritional status, and critical illness. Consequently, developmental physiology must be incorporated into dose selection rather than treated as a secondary covariate [45].

Unlike conventional pharmacologic agents that seek to maximize systemic exposure, the objective of OHB-607 was designed to restore circulating IGF-1 concentrations toward the physiologic fetal range that normally persists during late gestation but declines rapidly following extremely preterm birth [28,36,38]. Population pharmacokinetic modeling therefore integrated developmental physiology with early clinical pharmacokinetic data to define infusion regimens capable of maintaining gestational age-appropriate IGF-1 exposure [28,36,37,38,45].

Clinical studies demonstrated that continuous intravenous infusion of the rhIGF-1/rhIGFBP-3 complex produced predictable, dose-dependent increases in circulating IGF-1 concentrations [37,38]. Because free IGF-1 has a short circulating half-life, complexing IGF-1 with IGFBP-3 and administering it by continuous infusion was associated with relatively stable systemic exposure that more closely approximated endogenous fetal physiology while minimizing fluctuations in circulating concentrations [31,36,38].

This exposure-based strategy directly informed clinical dose selection. Rather than following traditional dose-escalation principles, dosing was guided by pharmacokinetic target attainment to restore physiologic IGF-1 concentrations while establishing pharmacokinetic behavior and short-term safety [28,36,38]. Population pharmacokinetic modeling identified infusion regimens capable of maintaining the desired physiologic exposure throughout the treatment period, and supported subsequent clinical evaluation [28,37,45].

Successful translation also required demonstration that prolonged continuous infusion could be integrated into routine neonatal intensive care. Analytical compatibility studies demonstrated preservation of protein integrity and physicochemical compatibility of rhIGF-1/rhIGFBP-3 with commonly administered neonatal medications, parenteral nutrition formulations, lipid emulsions, heparin, and crystalloid solutions, supporting formulation stability during routine NICU administration [46,47].

Practical implementation was further facilitated by integration with established neonatal vascular access practices designed to minimize line-related complications [48]. Because extremely premature infants routinely require central venous access for parenteral nutrition and intensive supportive care, prolonged rhIGF-1/rhIGFBP-3 infusion was intended to utilize existing vascular access strategies without increasing procedural burden [37]. Standardized catheter insertion and maintenance bundles have substantially reduced not only central line-associated bloodstream infections (CLABSIs) but complications associated with malposition of central lines, including complications of extravasation and perforation, thus providing the framework for safe administration of continuous infusion therapy [49]. Early clinical studies suggested that rhIGF-1/rhIGFBP-3 could be incorporated into routine NICU practice using established infection-prevention measures, standardized vascular access protocols, and flexible treatment approaches tailored to each infant’s evolving clinical condition [36,37,44].

Collectively, these studies suggest that successful translation of developmental therapeutics requires consideration of pharmaceutical compatibility, developmental pharmacology, vascular access management, and integration into routine neonatal intensive care in addition to biologic rationale. The OHB-607 development program provides one example of how these complementary elements can be incorporated into early clinical development. Whether similar approaches will facilitate broader application of developmental replacement therapies remains to be established through clinical investigation.

## 5. Evolution of BPD Severity Definitions and Clinical Trial Endpoints

Although bronchopulmonary dysplasia (BPD) is the most common chronic respiratory complication of extreme prematurity, defining clinically meaningful disease severity has evolved considerably over the past two decades [5]. The 2001 National Institute of Child Health and Human Development (NICHD) consensus definition classified BPD according to oxygen requirement at 36 weeks’ postmenstrual age (PMA) [6] and remains widely used in clinical trials. Advances in neonatal respiratory care subsequently prompted development of updated classifications, including the 2018 NIH workshop definition [50] and, more recently, the Jensen classification, which defines disease severity according to the mode of respiratory support at 36 weeks’ PMA, independent of oxygen concentration [51]. Compared with earlier oxygen-based definitions, the Jensen classification has been shown to more accurately predict death and serious respiratory morbidity during early childhood and has therefore been increasingly adopted in neonatal clinical trials and observational studies [51]. More recent studies suggest that contemporary BPD severity classifications have broadly similar ability to predict later respiratory and neurodevelopmental outcomes at 2 and 5 years of age [52,53].

The evolution of BPD classification has important implications for therapeutic development and clinical trial design. Contemporary trials increasingly evaluate prevention of severe BPD or use composite endpoints incorporating death before 36 weeks’ PMA, recognizing mortality as a competing outcome among the most immature infants. Consistent with this approach, the OHB-607 clinical development program evaluates severe BPD or death at 36 weeks’ PMA using a modified NICHD severity classification as the primary endpoint, with the Jensen (NRN) definition as a key secondary endpoint [37,38,51]. This approach reflects current practice in neonatal clinical trial design by incorporating both established and contemporary measures of clinically meaningful respiratory outcome rather than implying superiority of any single classification.

## 6. Discussion

Contemporary neonatal care has transformed survival of extremely preterm infants, yet BPD remains one of the most important unresolved complications of extreme prematurity despite major advances in neonatal intensive care, and its incidence has remained stable or increased as survival of extremely preterm infants has improved [1,3,4,5]. Contemporary management is largely supportive, minimizing injury and treating symptoms of BPD rather than correcting the developmental disturbances initiated by premature birth [5,16,17,18,19]. Physiologic IGF-1 replacement has been proposed as a complementary therapeutic strategy intended to restore one of the earliest developmental signals interrupted by separation from the maternal–placental environment during a critical period of pulmonary development. In this respect, IGF-1 replacement builds on the broader concept of correcting a physiologic deficiency, while targeting a broader developmental pathway regulating pulmonary vascular development, alveolarization, and coordinated maturation across multiple organ systems [36,38,42,54,55].

**Figure 1:**
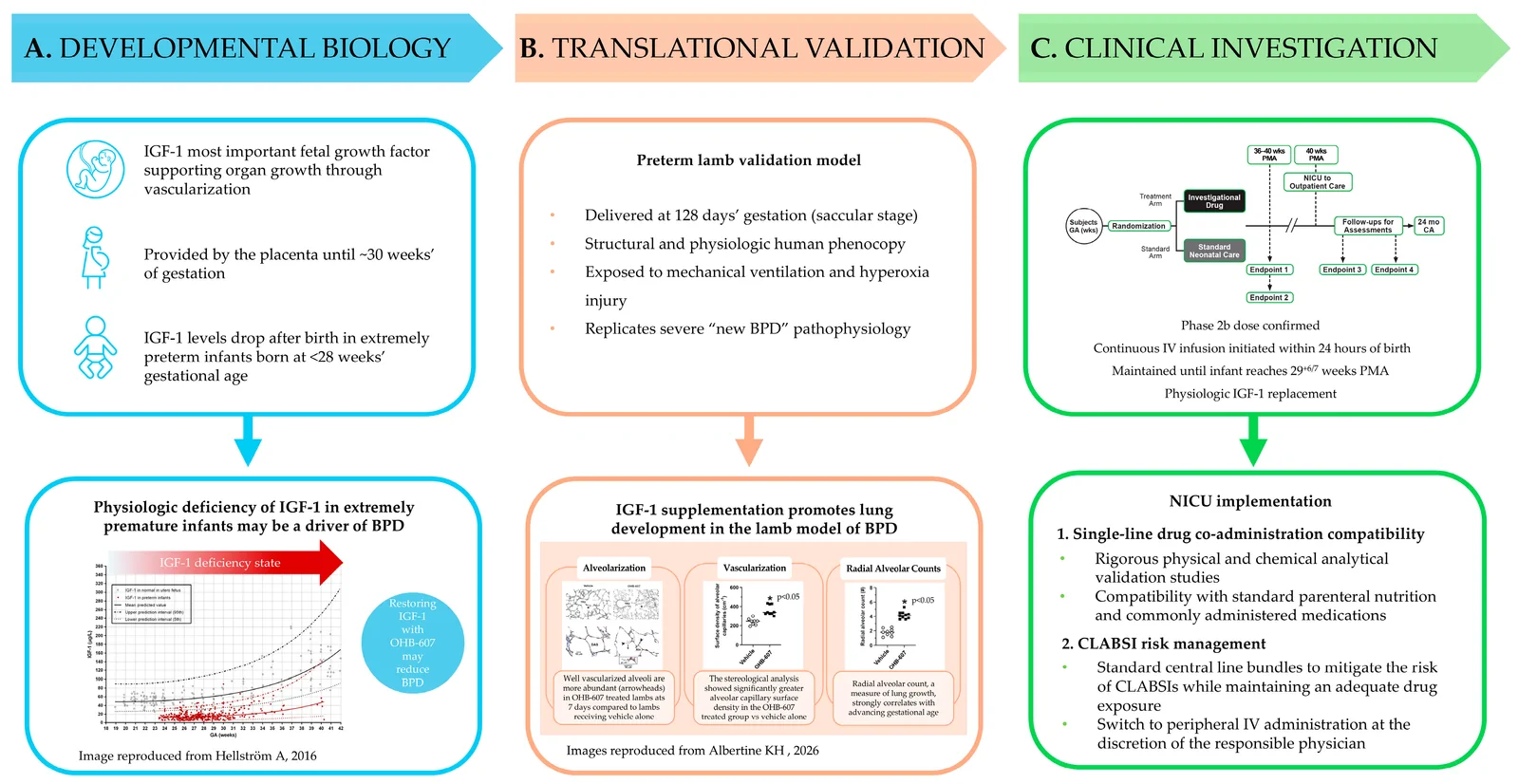
Translational development pathway supporting the clinical investigation of OHB-607. Premature birth is associated with an abrupt decline in circulating IGF-1 concentrations during a critical period of lung development (Panel (**A**)). Mechanistic studies and preterm lamb models provide translational evidence supporting investigation of physiologic IGF-1 replacement (Panel (**B**)). These findings informed the design and clinical implementation of the ongoing Phase 2b evaluation of OHB-607 (Panel (**C**)). Preclinical findings provide mechanistic and translational support but do not establish clinical efficacy, which requires confirmation in adequately powered randomized clinical trials [34,35,37,38,39,47,49]. BPD, bronchopulmonary dysplasia; CA, corrected age; CLABSI, central line-associated bloodstream infection; GA, gestational age; IGF-1, insulin-like growth factor 1; IV, intravenous; mo, month; PMA, postmenstrual age; wk, week.

The development of OHB-607 provides one example of how developmental biology may be translated into clinical investigation (Figure 1). Mechanistic studies, translational animal models, developmental pharmacology, and pharmaceutical development collectively support evaluation of physiologic IGF-1 replacement as one potential developmental strategy [30,31,32,34,35,38]. However, whether restoration of physiologic IGF-1 concentrations improves clinically meaningful pulmonary or multisystem outcomes remains uncertain and requires confirmation in adequately powered randomized clinical trials. The development program also examines the hypothesis that developmental replacement therapy may depend on restoring physiologic exposure rather than maximizing pharmacologic dose. Whether this principle applies broadly across neonatal therapeutics remains to be established [29,30,36,56].

Current clinical trials increasingly evaluate severe BPD or composite endpoints incorporating death, recognizing mortality as a competing outcome among the most immature infants [5,50,51,52,53]. Respiratory support-based classifications, including the modified NICHD and Jensen definitions, provide clinically meaningful measures that complement traditional oxygen-based definitions and more accurately predict longer-term respiratory outcomes [50,51,52,53]. Consistent with this evolution, the OHB-607 clinical development program incorporates severe BPD or death as the primary endpoint using a modified NICHD definition, with the Jensen classification as a key secondary endpoint [37,38]. Integrating developmental pharmacology with clinically meaningful severity-based endpoints may offer a useful approach for evaluating future neonatal developmental therapeutics [56].

Physiologic IGF-1 replacement should be viewed as complementary to contemporary neonatal intensive care rather than a substitute for established therapies. Although the translational evidence supporting IGF-1 biology is substantial, the clinical evidence remains limited. Most data currently derive from mechanistic investigations, animal models, pharmacokinetic studies, and early-phase clinical trials. Whether restoration of physiologic IGF-1 concentrations results in meaningful improvements in pulmonary or multisystem outcomes remains to be demonstrated in confirmatory randomized studies.

Because IGF-1 is a pleiotropic developmental regulator, postnatal deficiency may contribute not only to impaired pulmonary development but also to ROP, IVH, NEC, impaired somatic growth, and more broadly impaired brain growth and development [28,29,36,37,38,39,43,44,57]. Although these observations provide a biologically plausible basis for continued investigation, whether physiologic replacement improves multisystem outcomes remains to be established in adequately powered randomized clinical trials.

Several limitations should be acknowledged. Promising findings in experimental models do not necessarily translate into clinical benefit in the heterogeneous population of extremely preterm infants [58,59]. Responses to therapy may differ according to gestational age, fetal growth restriction, antenatal exposures, nutritional status, and severity of neonatal illness or even maternal illnesses. Although persistent postnatal IGF-1 deficiency is consistently associated with adverse outcomes, it may function as a causal mediator of impaired development, a biomarker of developmental immaturity, or an integrated indicator of nutritional status and systemic illness [29,39,43]. Restoration of a single developmental pathway is unlikely to fully reproduce the complex maternal–placental environment that orchestrates fetal growth and organ maturation. Accordingly, physiologic IGF-1 replacement should be viewed as one component of a broader developmental therapeutic strategy that complements, rather than replaces, contemporary neonatal intensive care [23,36].

Additional uncertainties also warrant consideration. Although IGF-1 has important developmental effects across multiple organ systems, long-term safety following physiologic replacement remains to be established. Likewise, whether early restoration of circulating IGF-1 translates into sustained improvements in pulmonary function, neurodevelopment, or overall health beyond infancy remains to be seen, and long-term follow-up is required. Extremely preterm infants also represent a highly heterogeneous population with substantial variability in gestational age, fetal growth, illness severity, nutritional status, and comorbidities that may influence both developmental biology and therapeutic response. The available clinical experience is relatively limited compared with established neonatal therapies, and uncommon adverse events may only become apparent through larger confirmatory studies and post-marketing surveillance should the therapy ultimately be approved, emphasizing the importance of adequately powered randomized trials with long-term follow-up. Finally, the authors acknowledge the potential for publication and interpretative bias in the presentation of translational evidence for OHB-607, particularly while definitive clinical trial results remain pending. OHB-607 is one of several emerging approaches to the prevention or treatment of BPD, alongside mesenchymal stromal cells (MSCs), MSC-derived extracellular vesicles, and intratracheal replacement of surfactant protein D combined with surfactant [60,61,62,63,64]. The coming decades are likely to yield a broader range of therapies targeting the biological pathways that contribute to BPD.

## 7. Conclusions

Despite remarkable advances in neonatal care, BPD remains a major unresolved consequence of extreme prematurity because current therapies primarily reduce secondary injury rather than target disrupted developmental processes. Developmental therapeutics has emerged as one potential complementary strategy to restore physiologic signaling pathways interrupted by premature birth. Among these, the IGF axis is one of the best-characterized developmental pathways linking pulmonary vascular development, alveolarization, and coordinated maturation across multiple organ systems. Experimental biology, translational animal studies, developmental pharmacology, and early clinical investigations collectively support continued evaluation of physiologic IGF-1 replacement as a mechanism-based therapeutic strategy [29,30,32,34,35,42,56,58,59].

The translational development of OHB-607 provided one example of how developmental biology can be systematically integrated with quantitative developmental pharmacology, pharmaceutical science, and clinically meaningful outcome measures to advance neonatal drug development. Whether physiologic IGF-1 replacement is associated with reductions in severe BPD, long-term respiratory health, neurodevelopment, and other clinically important outcomes remains to be established in adequately powered randomized clinical trials [37].

More broadly, the OHB-607 program illustrates one potential approach to developmental therapeutics in neonatology. By integrating developmental biology, translational science, developmental pharmacology, and practical implementation within the neonatal intensive care environment, this approach may inform the evaluation of other mechanism-based neonatal therapies. Whether similar developmental replacement strategies will prove effective for other disrupted signaling pathways remains an open question requiring further investigation.

## Figures and Tables

**Table 1:** Translational evidence supporting the clinical investigation of physiologic IGF-1 replacement. Experimental findings from animal models provide mechanistic and translational support but do not establish clinical efficacy. Confirmation of clinical benefit requires adequately powered randomized clinical trials. PD: pharmacodynamic; PK: pharmacokinetic.

Model	Objective	Intervention	Principal Findings	Contribution to Clinical Translation
Rodent developmental model [32,33]	Define the role of IGF-1 signaling in lung development	Genetic and pharmacologic modulation of IGF-1 signaling	Experimental modulation of IGF-1 signaling was associated with preservation of alveolarization, pulmonary vascular development, and extracellular matrix remodeling in models of BPD	Supported the biologic plausibility of IGF-1 replacement as a developmental therapeutic strategy
Premature lamb [34,35]	Evaluate physiologic IGF-1 replacement in a clinically relevant model of extreme prematurity	Continuous intravenous rhIGF-1/rhIGFBP-3 infusion during postnatal IGF-1 deficiency	Continuous infusion was associated with improvements in lung compliance, gas exchange, alveolarization, pulmonary vascular development, and reduced pulmonary hypertension	Supported translational investigation and progression to clinical evaluation
PK/PD modeling [31,38]	Define an exposure strategy to approximate fetal physiologic IGF-1 concentrations in extremely preterm infants	Population pharmacokinetic modeling and exposure-guided dose optimization	Early continuous infusion achieved gestational age-appropriate circulating IGF-1 concentrations	Supported dose selection and exposure-guided evaluation in Phase 2 clinical studies

## Data Availability

No new data were created or analyzed in this study. Data sharing is not applicable to this article.

## Abbreviations

The following abbreviations are used in this manuscript:

BPD	Bronchopulmonary dysplasia
CA	Corrected age
CLABSI	Central line-associated bloodstream infection
FDA	U.S. Food and Drug Administration
GA	Gestational age
IGF-1	Insulin-like growth factor 1
IGFBP-3	Insulin-like growth factor binding protein-3
IV	Intravenous
IVH	Intraventricular hemorrhage
NEC	Necrotizing enterocolitis
NICHD	National Institute of Child Health and Human Development
NICU	Neonatal intensive care unit
NRN	Neonatal Research Network
PD	Pharmacodynamic
PK	Pharmacokinetic
PMA	Postmenstrual age
rhIGF-1	Recombinant human insulin-like growth factor 1
rhIGFBP-3	Recombinant human insulin-like growth factor binding protein-3
VEGF	Vascular endothelial growth factor
